# Total Glucosides of *Paeonia lactiflora* for Safely Reducing Disease Activity in Systemic Lupus Erythematosus: A Systematic Review and Meta-Analysis

**DOI:** 10.3389/fphar.2022.834947

**Published:** 2022-01-31

**Authors:** Yifan Chen, Liuding Wang, Yu Cao, Nannan Li

**Affiliations:** Xiyuan Hospital, China Academy of Chinese Medical Sciences, Beijing, China

**Keywords:** total glucosides of paeony, systemic lupus erythematosus, meta-analysis, disease activity, safety

## Abstract

**Background:** Total glucosides of paeony (TGP), extracted from the dried roots of *Paeonia lactiflora* Pall., are proven to regulate immune function in various rheumatic diseases. We aim to systematically evaluate the efficacy and safety of TGP in reducing disease activity in systemic lupus erythematosus (SLE).

**Methods:** We searched trials in seven electronic databases and two clinical trail registries. Randomized controlled trials (RCTs) evaluating efficacy and safety of TGP for SLE were identified. The Cochrane Risk of Bias Tool 2.0 was used for quality assessment of the included trials, and RevMan 5.4 software was used for meta-analysis.

**Results:** A total of 14 RCTs were included, including 978 participants, 492 in the intervention group and 486 in the control group. Regarding the efficacy of TGP for SLE, results showed that TGP plus conventional treatments (CTs) was superior to CTs alone in reducing disease activity (*MD*_SLEDAI-1m_ = −3.54, 95% *CI* = −4.08 to −3.00, *p* < 0.00001; *MD*_SLEDAI-2m_ = −3.80, 95% *CI* = −4.51 to −3.09, *p* < 0.00001; *MD*_SLEDAI-3m_ = −1.62, 95% *CI* = −2.60 to −0.64, *p* < 0.0001; *MD*_SLEDAI-6m_ = −1.97, 95% *CI* = −3.18 to −0.76, *p* = 0.001). The results also showed that TGP contributed to a betterment in improving other outcomes related to lupus activity, such as ESR, CRP, complement proteins (C3, C4), and immunoglobulins (IgA, IgM). In addition, TGP significantly decreased average daily glucocorticoid dosage and cumulative cyclophosamide dosage, as well as disease recurrence rate. In terms of safety, TGP may reduce the incidence of adverse reactions (*RR* = 0.51, 95% *CI* = 0.29 to 0.88, *p* = 0.01). The certainty of the evidence were assessed as moderate to low.

**Conclusion:** TGP appears potentially effective and generally safe in reducing disease activity in SLE. However, in view of high risk of bias, the findings need to be confirmed in high-quality trials.

**Systematic Review Registration**: https://www.crd.york.ac.uk/prospero, identifier CRD42021274850

## Introduction

Systemic lupus erythematosus (SLE, ICD10 Code: M32.9) is an autoimmune disorder progressively resulting in multi-system organ damage (Piga and Arnaud, 2021). Compared with other rheumatic diseases, the irreversible multiorgan involvement and dysfunction of SLE lead to more life-threatening complications, including infections, renal failure, pulmonary arterial hypertension and cardio-cerebrovascular diseases (Mu et al., 2018). Moreover, for patients who entered the early quiescent state of the disease, there was still a 60% risk of subsequent flare (Nossent et al., 2010), which as well as the more treatment resources needed was important factor resulting in a substantial disease burden (Jönsen et al., 2015).

Up to now, hydroxychloroquine, glucocorticoids and immunosuppressants have been recommended treatments for SLE (Tunnicliffe et al., 2015). Despite the improved prognosis with the emergence and appliance of these therapies (Lisnevskaia et al., 2014), numerous adverse reactions of all the above drugs cause worrisome comorbidities, covered by retinal toxicity, fertility failure, et cetera. A case-control study reported that 5.5% of patients exposed to antimalarial drugs developed anti-malarial retinal complications over an average 12.8 years of follow-up (Mukwikwi et al., 2020). In one retrospective study a higher cumulative cyclophosphamide dose was more prone to be premature ovarian failure (Sen et al., 2021). In addition, emerging studies have suggested that the use of glucocorticoid in SLE actually contributed to some harmful outcomes (Apostolopoulos and Morand, 2016; Kasturi and Sammaritano, 2016). High cumulative corticosteroid dose and immunosuppressant use increased risk for avascular necrosis and herpes zoster (Hu et al., 2016; Chen et al., 2017; Kwon et al., 2018). Hence, there is still no optimal therapeutic scheme defined to safely control disease activity and reduce the total costs (Jönsen et al., 2015).

**FIGURE 1:**
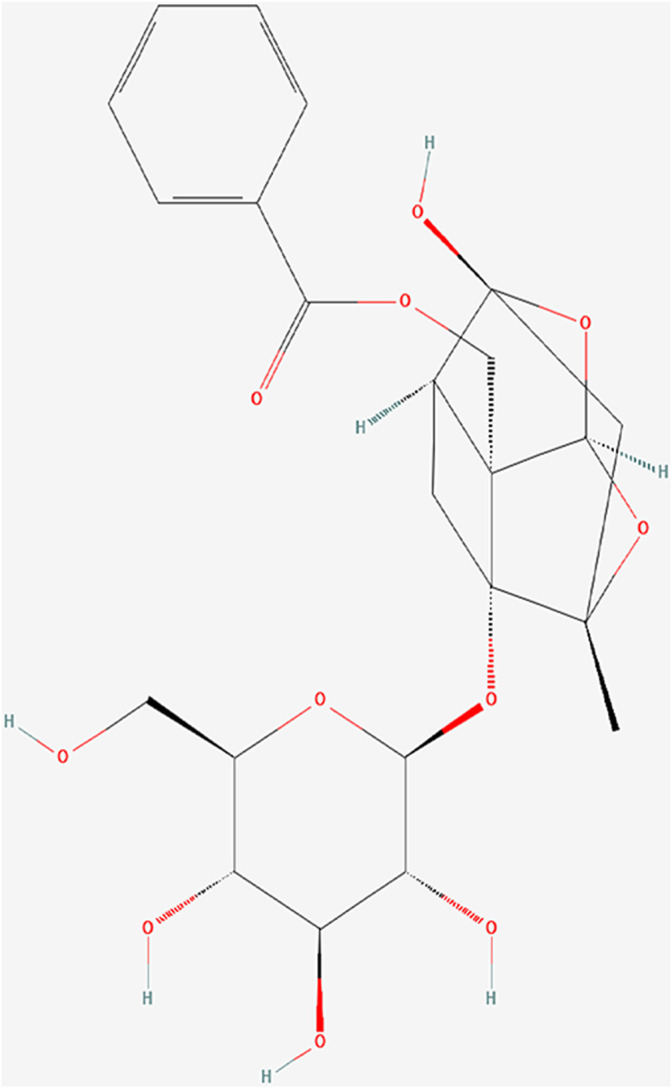
PubChem identifier: Paeoniflorin.

In China, Total glucosides of paeony (TGP), an ethanol-water extract of dried roots of *Paeonia lactiflora* Pall. (Baishao in Chinese), has been successfully applied in clinical treatment of autoimmune diseases, such as rheumatoid arthritis (Huang et al., 2019a), primary Sjögren’ s syndrome (Feng et al., 2019) and ankylosing spondylitis (Huang et al., 2019b). Paeoniflorin (Pae) (Figure 1; PubChem Identifier: Paeoniflorin; URL: https://pubchem.ncbi.nlm.nih.gov/compound/442534#section=2D-Structure), a water-soluble monoterpene glucoside, is the predominant constituent of TGP (Zhang and Wei, 2020). Previous studies have confirmed its various pharmacological effects, including immunoregulatory, anti-inflammatory, antioxidant and anti-organ-damage (Jiang et al., 2020; Zhang and Wei, 2020). Some further investigation in rat models and patients of SLE have revealed the mechanism that TGP inhibited autoimmunity possibly by downregulating ERα expression (Li and Jiang, 2019), inhibiting the IRAK1-NF-κB pathway (Ji et al., 2018), and enhancing DNA methylation of ITGAL promoter in CD4 (+) T cells (Zhao et al., 2012).

Currently, no study has followed the Preferred Reporting Items for Systematic Review and Meta-Analyses (PRISMA) statement to evaluate the efficacy and safety of TGP for SLE. There is a lack of robust evidence regarding reducing disease activity of TGP for SLE. It is known that lupus high disease activity state is closely assosiated with high mortality and economical burden (Polachek et al., 2017; Zen et al., 2017). And treatment recommendations are focusing on controlling disease activity and minimizing comorbidities (van Vollenhoven et al., 2014). Given the severity of SLE and clinical significance of disease activity, our study aimed to investigate the efficacy of TGP on safely reducing disease activity in patients with SLE.

## Methods

### Protocol Register

This systematic review and meta-analysis followed the PRISMA statement (Page et al., 2021). The review protocol was registered at PROSPERO (Registration number: CRD42021274850).

### Search Strategy

PubMed, embase, Cochrane Library, China National Knowledge Infrastructure (CNKI), Wanfang Database, SinoMed, the China Science Technology Journal Database (VIP) were searched from their inception to 1 March 2021. We also performed a comprehensive search of two clinical trail registries, ClinicalTrials.gov and Chinese Clinical Trial Register. The detailed search strategies of all databases are presented in Supplementary Table S1.

On 16 November 2021, We updated the database search of Pubmed and CNKI. We used the same search method, except that we narrowed the searches to March 2021 onwards.

### Inclusion Criteria

#### Types of Studies

We included randomized controlled trials (RCTs).

#### Types of Participants

Patients were diagnosed with SLE according to any recognized criteria, and at active phase. There was no limitation in age, gender and course of disease.

#### Types of Interventions

Intervention groups were treated with TGP plus conventional treatments (CTs), while control groups were treated with the CTs. Referring to the EULAR-SLE guidelines and the Chinese guidelines for the management of SLE (Fanouriakis et al., 2019; Chinese Rheumatology Association, 2020), CTs include hydroxychloroquine, glucocorticoids, immunosuppressive drugs and biological agents.

#### Types of Outcomes

Efficacy outcomes: The primary outcome is the Systemic Lupus Erythematosus Disease Activity Index (SLEDAI) score, including the SLEDAI-2K (Gladman et al., 2002) and the original SLEDAI (Bombardier et al., 1992), and the secondary outcomes are erythrocyte sedimentation rate (ESR), C-reactive protein (CRP), 24-h urine protein, complement proteins (C3, C4), immunoglobulins (IgA, IgM, IgG), average daily glucocorticoid dosage, cumulative cyclophosphamide dosage, and disease recurrence rate.

Safety outcomes: Incidence of adverse reactions and adverse events.

### Exclusion Criteria

We excluded trials as follows: 1) other traditional Chinese medicine treatments were applied in either intervention or control group; 2) trials with duplicate publications, data errors, and unavailability of full text; and 3) language is not Chinese or English.

### Study Selection

Two reviewers (YFC and LW) independently performed literature selection according to the predefined eligibility criteria. The records searched were imported into NoteExpress 3.2, and the duplicates were removed. Records were first screened based on the titles and abstracts, and in cases of uncertainty, the full texts were obtained. Any disagreement between the paired reviewers was resolved through discussing with a third reviewer (YC).

### Data Extraction

The following data were extracted from each trial by two reviewers (YFC and LDW):

1) identification information (the first author, and year of publication); 2) study designs (sample sizes, methods of randomization and allocation concealment, and details of blinding, et al.); 3) baseline characteristics of participants; 4) details of intervention and control groups; and 5) outcomes (dichotomous data were number of events and total participants per group; continuous data were presented as mean, standard deviation, and total participants per group).

Discrepancies were solved by discussion between two reviewers or arbitrated by the third researcher (YC) if necessary.

### Risk of Bias Assessment

Two reviewers (YFC and LW) independently assessed the risk of bias of the included trials. Using the Cochrane risk of bias tool 2.0 (Sterne et al., 2019), five domains were evaluated as follows: randomization process, deviations from the intended interventions, missing outcome data, measurement of the outcome and selection of the reported result. Each domain was ranked as “low risk of bias,” “some concerns,” or “high risk of bias.” If disagreements on the assessment were identified, the third author (YC) was consulted.

### Data Analysis

Review Manager 5.4 (RevMan 5.4) software was utilized to conduct the data analysis of dichotomous and continuous outcomes, which were extracted from the primary trials. Risk ratio (*RR*) was used for dichotomous data while weighted mean difference (*WMD*) or standardized mean difference (*SMD*) were adopted for continuous data as effect size, both of which were demonstrated with effect size and 95% confidence intervals (*CI*). When no statistical heterogeneity was identified (heterogeneity test, *p* ≥ 0.10, or *I* ^*2*^ ≤ 50%), a fixed-effects model was selected, otherwise a random-effects model was applied.

We performed subgroup analyses based on the course of treatment, or follow-up time. Sources of heterogeneity would be fully explored if enough data were available. We would conduct sensitivity analysis, sequently omitting each study. If the statistical heterogeneity changed significantly after studies were excluded, re-read the full texts further, focusing the information that may lead to clinical heterogeneity and methodological heterogeneity.

### Reporting Bias Assessment

To assess small-study effects, we planned to generate funnel plots for meta-analyses including at least ten trials of varying size to detect the publication bias. And we performed Begg’s rank correlation and Egger’s linear regression tests to assess the symmetry of funnel plot. To assess outcome reporting bias, we compared the outcomes specified in trial protocols with the outcomes reported in the corresponding trial publications; if trial protocols were unavailable, we compared the outcomes reported in the methods and results sections of the trial publications.

### Certainty Assessment

Two reviewers (YFC and LDW) independently assessed the certainty of the evidence using the Grading of Recommendations Assessment, Development and Evaluation (GRADE) approach (Guyatt et al., 2008; Balshem et al., 2011), and the evidence certainty were graded as “high,” “moderate,” “low,” or “very low.” The certainty can be downgraded for five GRADE considerations (study limitations, consistency of effect, imprecision, indirectness, and publication bias) and upgraded for three reasons (large magnitude of an effect, dose-response gradient, and effect of plausible residual confounding).

## Results

### Study Selection

**FIGURE 2:**
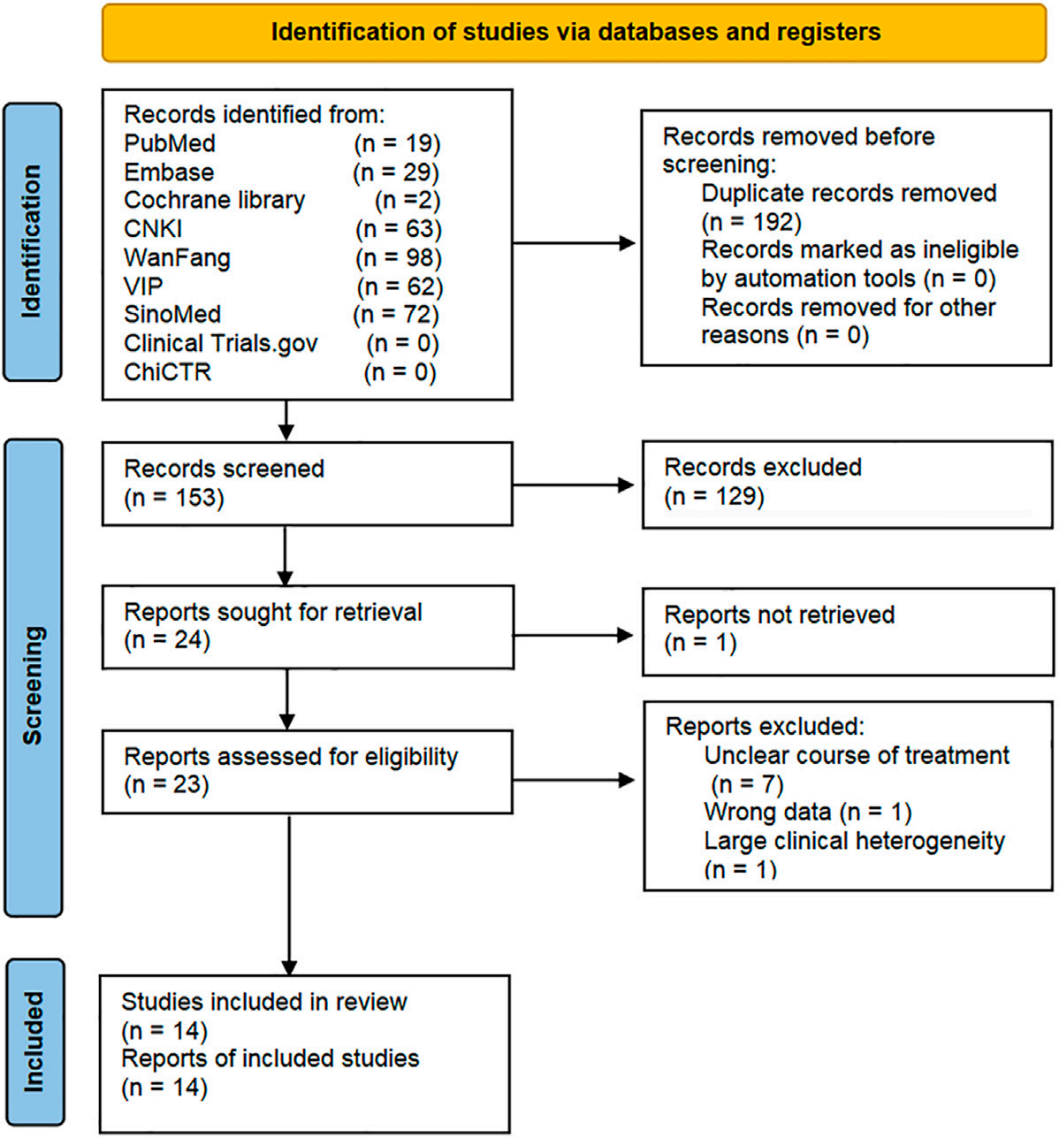
Flow diagram for identification of studies.

The initial search yielded 345 records, of which 192 were duplicate. After reading the titles and abstracts, the remaining 23 records were assesed by reading their full texts. There were nine trials removed, and the list of them is presented in Supplementary Table S2 with various reasons. No new trial was included after an updated retrieval and selection, up to 16 November 2021. Ultimately, 14 trials were included (Zhu and Wei, 2009; Sun, 2013; Wang and Wang, 2015; Xu, 2015; Cai et al., 2017; Li Z et al., 2018; Peng, 2018; Xue and Lyu, 2019; Yang and Li, 2019; Yu et al., 2019; Li and Zheng, 2020; Xiang, 2020; Zhang, 2020; Zhao et al., 2020). Literature screening process is shown in Figure 2.

### Study Characteristics

The general characteristics of the included trials are summarized in Table 1. A total of 14 RCTs involving 978 participants were included, all of which were Chinese literatures published between 2009 and 2020. The sample sizes varied from 40 to 106, with a total of 492 patients in the intervention group and 486 patients in the control group. The patients in intervention groups were treated with TGP plus CTs (prednison, prednisolone acetate, methylprednisolone, cyclophosphamide, tacrolimus, or hydroxychloroquine sulfate), and in control groups were treated with CTs alone. The course of treatment ranged from 1 month to 6 months. In 13 trials (Zhu and Wei, 2009; Sun, 2013; Wang and Wang, 2015; Xu, 2015; Cai et al., 2017; Li et al., 2018; Peng, 2018; Yang and Li, 2019; Yu et al., 2019; Li and Zheng, 2020; Xiang, 2020; Zhang, 2020; Zhao et al., 2020), SLEDAI score was reported as a primary outcome. And incidence of adverse reactions was reported in 10 trials (Wang and Wang, 2015; Xu, 2015; Cai et al., 2017; Li et al., 2018; Peng, 2018; Xue and Lyu, 2019; Yang and Li, 2019; Yu et al., 2019; Li and Zheng, 2020; Zhao et al., 2020). The source, quality control, and chemical characterisation of TGP used in the included trials are presented in Supplementary Table S3.

**TABLE 1:** Basic characteristics of included studies.

**Study**	**Sample size**		Male/Famale		Age/(year)		Intervention		Treatment duration	Outcomes
	T	C	T	C	T	C	T	C		
Zhu and Wei, 2009	35	30	−/−	−/−	16–55 (32 ± 6.5)	16–55 (32 ± 6.5)	TGP 0.6 g tid plus CTs	GC ≥ 1 mg/kg/day for severe activity, 0.5 mg/kg/day for mild to moderate activity, < 15 mg/day for no activity, and tapered according to the condition	3 months	1) 2) 3) 5) 7)
Sun (2013)	48	48	−/−	−/−	35.0 ± 0.17	35.0 ± 0.17	TGP 0.6 g tid plus CTs	GC 0.2 mg/kg/day; CTX 0.5 g once every 2 weeks	3 months	1) 4) 5) 6)
Wang and Wang (2015)	21	21	−/−	−/−	27.34 ± 7.65	29.28 ± 8.95	TGP 0.6 g tid plus CTs	GC 0.5–1 mg/kg/day, and then reduced by 10% weekly according to the condition	3 months	1) 10)
Xu (2015)	27	26	10/17	9/17	42.7 ± 5.3	46.2 ± 7.4	TGP 0.6 g tid plus CTs	GC 0.6–0.8 mg/kg/day for 6 weeks, then reduced 5 mg weekly until 30 mg/day, and then reduced 5 mg every 2 weeks until 5–10 mg/day; CTX 0.5 g for 2 days every half month (0.2 g for day 1, and 0.3 g for day 2), then adjusted it to every 1–6 months according to the condition, with the cumulative dose was 2–3 g	1 month	1) 4) 7) 8) 10)
Cai et al. (2017)	30	30	5/25	4/26	33.2 ± 5.1	34.7 ± 4.5	TGP 0.6 g tid plus CTs	GC 0.8 mg/kg/day for 1 week, then reduced 3 mg weekly until 20 mg/day; CTX 15 mg/kg once every 2 weeks, and after up to 5 consecutive treatments, adjusted it to once every 4 weeks	6 months	1) 2) 4) 5) 7) 8) 10)
Li Z et al., 2018	45	45	9/36	7/38	18–65 (32.15 ± 5.37)	19–65 (33.21 ± 4.94)	TGP 1.2 g bid plus CTs	GC; TAC 0.05 mg/kg/day bid for 6 months	6 months	1) 5) 6) 7) 9) 10)
Peng (2018)	20	20	4/16	6/14	43.3 ± 4.9	43.7 ± 4.6	TGP 0.6 g tid plus CTs	GC 0.9 mg/kg/day for 6 weeks, then reduced 5 mg weekly until 30 mg/day, and then reduced 5 mg every 2 weeks until 5–10 mg/day; CTX 1 g for 2 days every 15 days (0.4 g for day 1, and 0.6 g for day 2), then adjusted it to every 1–5 months according to the condition, with the cumulative dose of 5 g	2 months	1) 4) 7) 8) 9) 10)
Yang and Li, 2019	53	53	5/48	3/50	41.5 ± 4.1	42.7 ± 5.2	TGP 0.6 g tid plus CTs	GC 0.5–1 mg/kg/day, and then reduced by 10% weekly according to the condition	3 months	1) 10)
Xue and Lyu, 2019	30	30	3/27	3/27	38.15 ± 3.20	38.12 ± 3.25	TGP 0.6 g tid plus CTs	GC 15 mg qd; HCQ 0.2 g bid	6 months	2) 5) 10)
Yu et al., 2019	30	30	6/24	5/25	22–55 (37.84 ± 5.93)	23–56 (37.94 ± 5.82)	TGP 0.6 g bid/tid plus CTs	GC 0.5–1 mg/kg/day for 6 weeks, then reduced 5 mg weekly until 30 mg/day, and then reduced 5 mg every 2 weeks until 5–10 mg/day; CTX 0.4 g once every 2 weeks for 3 months, then adjusted it to 0.8–1 g every 4 weeks, and then adjusted it to every 3–6 months according to the condition, with the cumulative dose of 2–3 g	6 months	1) 2) 3) 5) 7) 8) 10)
Zhang (2020)	32	32	17/15	14/18	21–58 (38.2 ± 0.5)	23–61 (37.9 ± 1.2)	TGP 0.6 g bid plus CTs	GC 15–20 mg bid; TAC 0.05 mg/kg bid	6 months	1)
Zhao et al. (2020)	53	53	3/50	5/48	17–59 (38.14 ± 3.24)	15–60 (34.13 ± 3.22)	TGP 0.6 g tid plus CTs	GC 4 mg bid for 4 weeks, then 4 mg qd for 4 weeks, and then reduced to 2 mg qd; HCQ 0.2 g bid	3 months	1) 5) 10)
Xiang (2020)	35	35	9/26	8/27	23–58 (39.2 ± 4.3)	21–57 (38.5 ± 4.2)	TGP 0.6 g bid/tid plus CTs	GC 0.5–1 mg/kg/day for 6 weeks, then reduced 5 mg weekly until 30 mg/day; CTX 0.4 g once every 2 weeks for 3 months, then adjusted it to 0.8–1 g every 3–6 months according to the condition, with the cumulative dose of 2–3 g	6 months	1) 7) 8)
Li and Zheng, 2020	33	33	8/25	6/27	20–71 (47.21 ± 7.42)	22–67 (44.52 ± 7.37)	TGP 0.6 g bid plus CTs	GC 0.8 mg/kg/day for 6 weeks, then reduced 5 mg weekly until 30 mg/day, and then reduced 5 mg every 2 weeks until 5–10 mg/day; CTX 0.6 g once every 2 weeks for 3 months, then 0.6 g monthly for 3 months, and then adjusted it to once every 3 months	1 month	1) 4) 7) 8) 9) 10)

C, control group; CTX, cyclophosphamide; GC, glucocorticoid; HCQ, hydroxychloroquine sulfate; TAC, tacrolimus; T, intervention group.

Outcomes: 1) SLEDAI, score; 2) ESR; 3) CRP; 4) 24-h total urine protein; 5) complement proteins (C3, C4) ; 6) immunoglobulins (IgA, IgM, IgG) ; 7) the average daily glucocorticoid dosage; 8) cumulative cyclophosphamide dosage; 9) disease recurrence rate; 10) incidence of adverse reactions; 11) adverse events.

### Assessment of Risk of Bias

**FIGURE 3:**
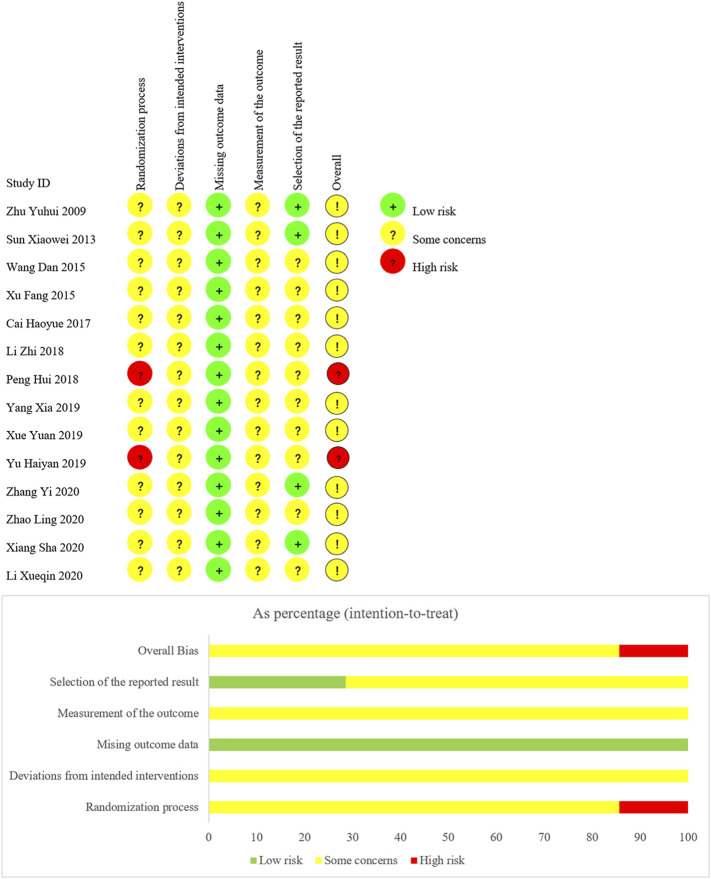
Risk of bias of included studies.

We have summarized risk of bias of included trials in Figure 3.

### Domain 1: Risk of Bias Arising From the Randomization Process

For random sequence generation, four trials (Cai et al., 2017; Li et al., 2018; Yang and Li, 2019; Zhao et al., 2020) used a random number table, one trail (Xue and Lyu, 2019) used coin toss randomization, one trial (Xiang, 2020) used ball touch method, two trails (Peng, 2018; Yu et al., 2019) used admission order, and the other six trials (Zhu and Wei, 2009; Sun, 2013; Wang and Wang, 2015; Xu, 2015; Li and Zheng, 2020; Zhang, 2020) were lack of describing their methods in generating random sequence. For allocation concealment, no trial reported information. Due to the insufficient information, we judged twelve trials as “some concerns,” and two trials (Peng, 2018; Yu et al., 2019) as “high risk of bias.”

### Domain 2: Risk of Bias Due to Deviations From the Intended Interventions

There was no trial reported whether blinding was implemented. Based on the available information, we were unable to accurately speculate the deviations from intended interventions. Therefore, we judged all trials as “some concerns.”

### Domain 3: Risk of Bias Due to Missing Outcome Data

All data of outcomes were available, so we judged all trials as “low risk of bias.”

### Domain 4: Risk of Bias in Measurement of the Outcome

There was no trial reported whether the assessors were blinded. Therefore, we judged all trials as “some concerns” in this domain.

### Domain 5: Risk of Bias in Selection of the Reported Result

Although no trial protocol was available, all trials fully reported the outcomes planned in the method section of published reports. However, there were ten trials (Wang and Wang, 2015; Xu, 2015; Cai et al., 2017; Li M et al., 2018; Peng, 2018; Xue and Lyu, 2019; Yang and Li, 2019; Yu et al., 2019; Li and Zheng, 2020; Zhao et al., 2020), only reporting adverse reactions, lacking the judgment on the possibility related to treatments. We suspected there were other unreported adverse events. Consequently, we judged the ten trials as “some concerns,” due to potential selective reporting bias. Other trials were judged as “low risk of bias.”

According to the assessment of above five domains, we judged the overall bias of two trials (Peng, 2018; Yu et al., 2019) as “high risk of bias,” and the overall bias of other trials as “some concerns.”

### Efficacy Outcomes

#### SLEDAI Score

**FIGURE 4:**
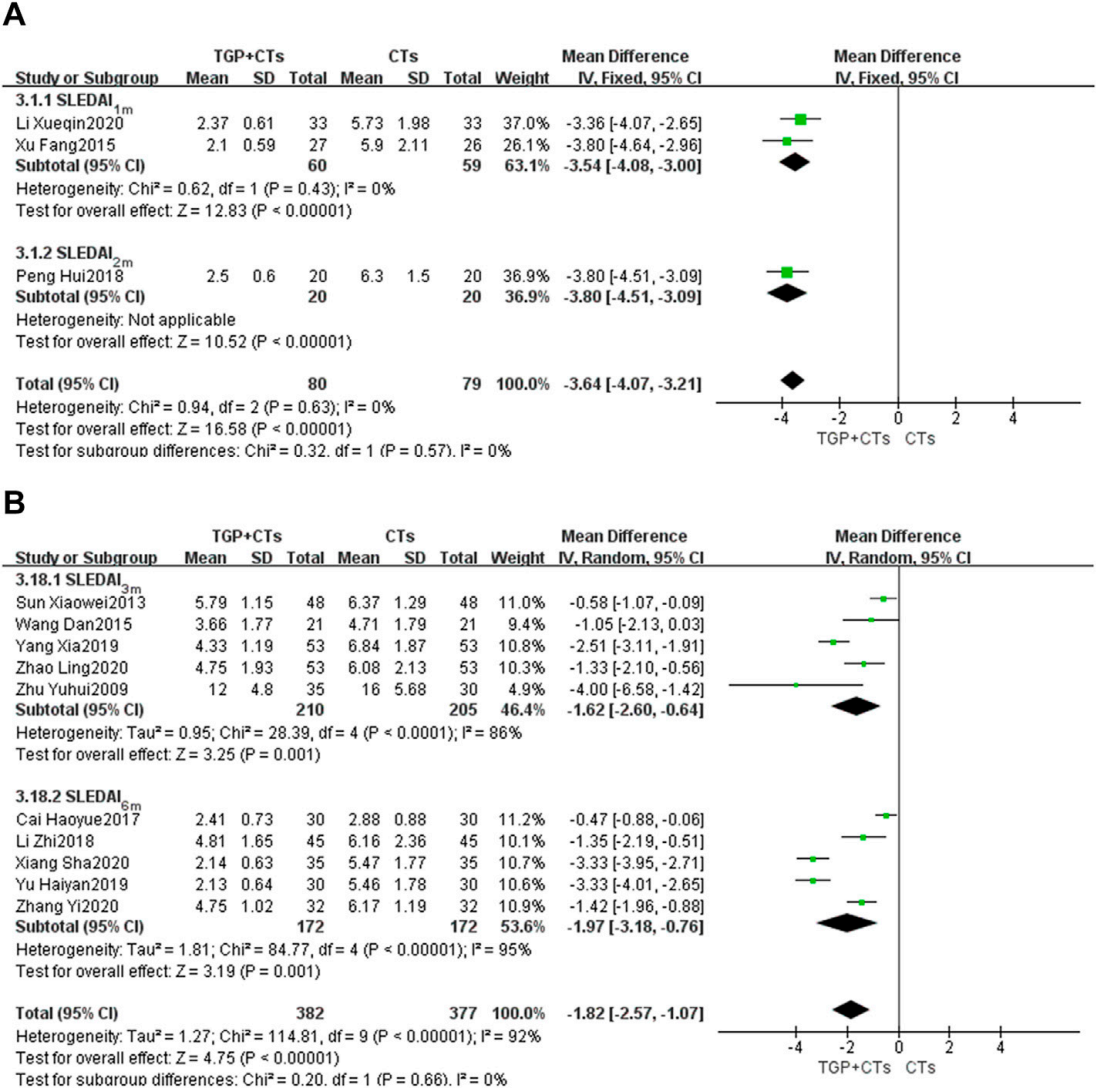
Forest plot of SLEDAI score at 1 month, 2 months **(A)**, 3 and 6 months **(B)**.

There were 13 trials reporting SLEDAI score as the primary outcome, and subgroup analysis was conducted according to the treatment duration. The duration of two trials (Xu, 2015; Li and Zheng, 2020) was 1 month, and one trial (Peng, 2018) was 2 months. We used the fixed effect model because of the insignificant heterogeneity (*P*_SLEDAI-1m_ = 0.43, *I* ^2^ = 0%). The results showed that the intervention group was superior to the control group in improving SLEDAI score (*MD*_SLEDAI-1m_ = −3.54, 95% *CI* = −4.08 to −3.00, *p* < 0.00001; *MD*_SLEDAI-2m_ = −3.80, 95% *CI* = −4.51 to −3.09, *p* < 0.00001; Figure 4A).

Five trials (Zhu and Wei, 2009; Sun, 2013; Wang and Wang, 2015; Yang and Li, 2019; Zhao et al., 2020) were treated for 3 months and the other five trials (Cai et al., 2017; Li et al., 2018; Yu et al., 2019; Xiang, 2020; Zhang, 2020) were treated for 6 months. We used the random-effect model according to the large heterogeneity (*P*_SLEDAI-3m_ < 0.0001, *I* ^2^ = 86%; *P*_SLEDAI-6m_ < 0.00001, *I* ^2^ = 95%). Results showed that the intervention group was better than the control group in reducing lupus activity, with statistically significant differences (*MD*_SLEDAI-3m_ = −1.62, 95% *CI* = −2.60 to −0.64, *p* = 0.0001; *MD*_SLEDAI-6m_ = −1.97, 95% *CI* = −3.18 to −0.76, *p* = 0.001; Figure 4B). However, the large heterogeneity affected the credibility of the results, we performed the sensitivity analysis to explore the sources of heterogeneity.

After the sequential exclusion of each trial and reading full texts, in the subgroup of 3 months, we found two trials (Zhu and Wei, 2009; Yang and Li, 2019) had a significant impact on the results, with heterogeneity decreasing (*p* = 0.25, *I* ^2^ = 28%). We eliminated the two trials and plooed other three trials (*MD*_SLEDAI-3m_ = -0.88, 95% *CI* = -1.37 to -0.39, *p* = 0.0004; Supplementary Figure S1). In initial disease activity, one trial (Zhu and Wei, 2009) was much higher than other trials, which may account for the partial heterogeneity.

#### ESR

**FIGURE 5:**
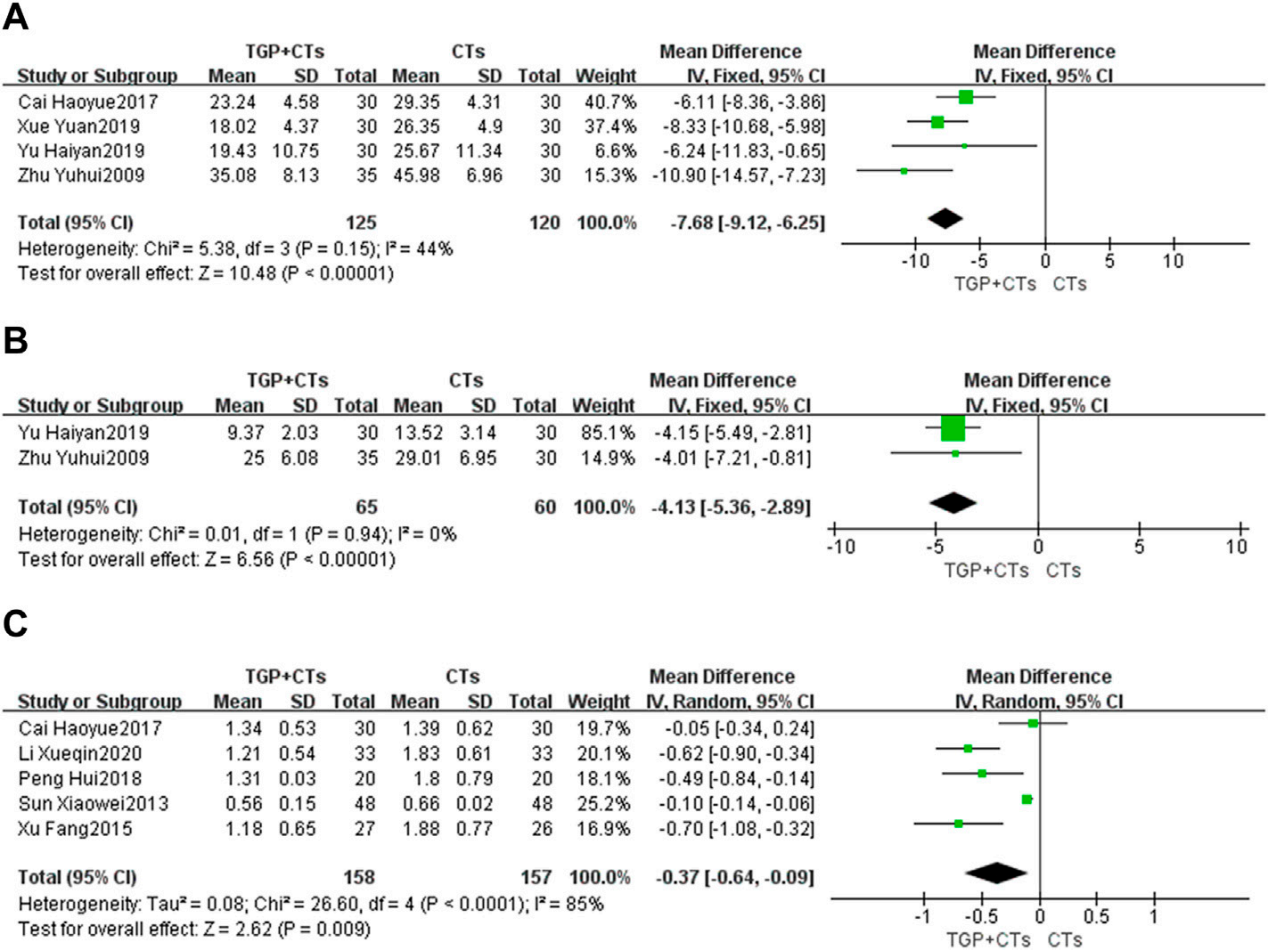
Forest plot of ESR **(A)**, CRP **(B)** and 24-h urine protein **(C)**.

Four trials reported ESR. There was no significant statistical heterogeneity (*p* = 0.15, *I* ^2^ = 44%), so fixed-effect model was used. The results showed that the intervention group was superior to the control group in reducing ESR (*MD* = −7.68, 95% *CI* = −9.12 to −6.25, *p* < 0.00001; Figure 5A).

#### CRP

Two trials reported CRP. The heterogeneity between them was insignificant (*p* = 0.94, *I* ^2^ = 0%), so the fixed-effect model was adopted. The results showed that the intervention group was superior to the control group in reducing CRP (*MD* = −4.13, 95% *CI* = −5.36 to −2.89, *p* < 0.00001; Figure 5B).

#### Tweny-Four Hour Urine Protein

Five trials reported 24-h urine protein. The heterogeneity (*p* < 0.0001, *I* ^2^ = 85%) was substantial, so we used the random effect model. The results showed that the intervention group was better than the control group in improving 24-h urine protein, and the difference was statistically significant (*MD* = -0.37, 95% *CI* = -0.64 to -0.09, *p* = 0.009; Figure 5C). Due to large heterogeneity, the source of heterogeneity was explored by conducting sensitivity analysis. It was found that the population age range of two trials (Sun, 2013; Cai et al., 2017) and the other three trials were different. In addition, the treatment duration of the five trials were different, so the above clinical heterogeneity may be the source of statistical heterogeneity. We removed the two trials, merging the rest three trials (*MD* = 0.60, 95% *CI* = 0.79 to 0.41, *p* < 0.00001; Supplementary Figure S2), heterogeneity decreased significantly (*p* = 0.72, *I* ^2^ = 0%).

#### Complement Proteins

**FIGURE 6:**
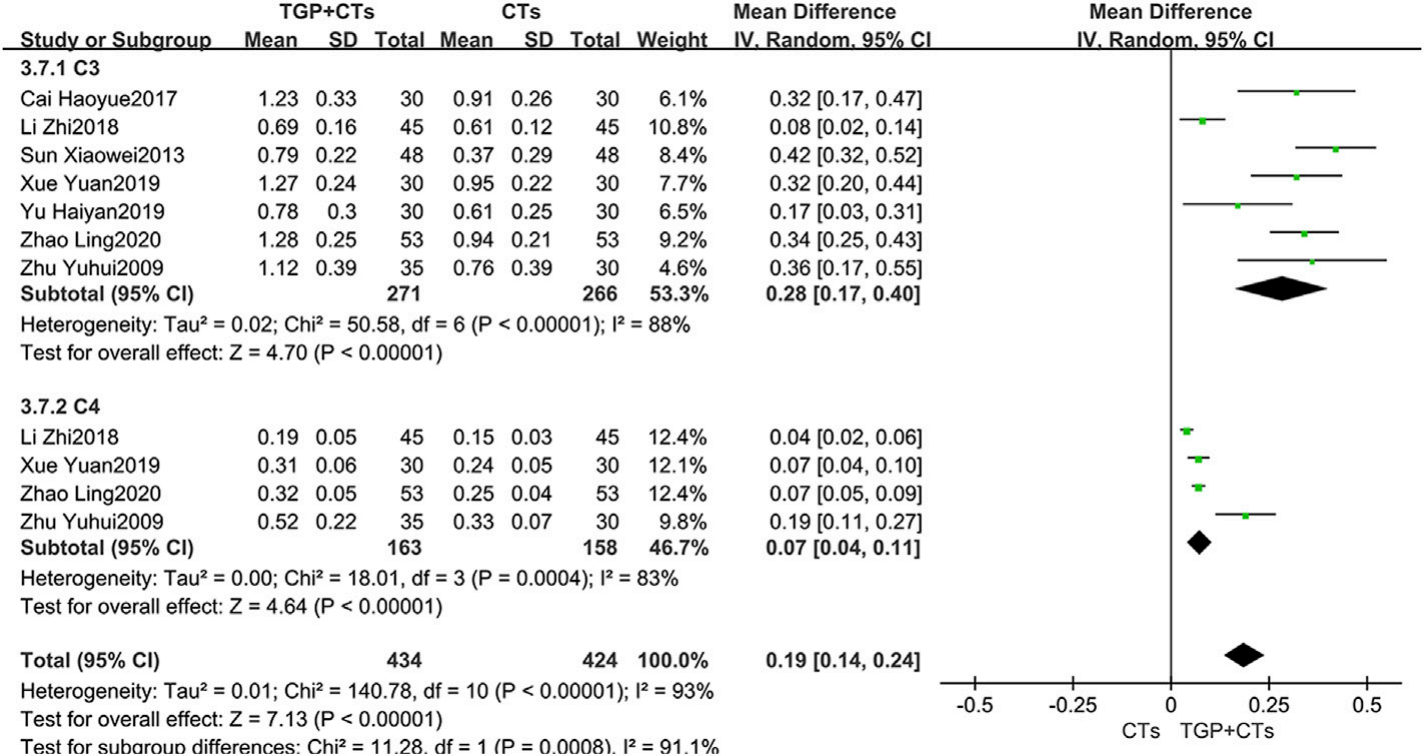
Forest plot of C3 and C4.

Seven trials reported C3 and 4 trials reported C4. Since the heterogeneity was large (*P*_C3_ < 0.00001, *I* ^2^ = 88%; *P*_C4_ = 0.0004, *I* ^2^ = 83%), the random effect model was used. The results suggested that the intervention group was superior to the control group in increasing C3 and C4, with statistical difference (*MD*_C3_ = 0.28, 95% *CI* = 0.17 to 0.40, *p* < 0.00001; *MD*_C4_ = 0.07, 95% *CI* = 0.04 to 0.11, *p* < 0.00001; Figure 6).

After sensitivity analysis and careful reading of original texts, in the subgroup of C3, it was found that the dosage and frequency of TGP in two trials (Li et al., 2018; Yu et al., 2019) were different from those in other five trials. Omitting the two trials, we pooled the other five trials alone (*MD*_C3_ = 0.36, 95% *CI* = 0.30 to 0.41, *p* < 0.00001; Supplementary Figure S3), and the heterogeneity was insignificant (*p* = 0.70, *I* ^2^ = 0%).

In the subgroup of C4, we found that the average age of participants of the two trials (Zhu and Wei, 2009; Li et al., 2018) was significantly different from that of the other two trials. We removed the two trials, and pooled the other two trials (*MD*_C4_ = 0.07, 95% *CI* = 0.06 to 0.08, *p* < 0.00001; Supplementary Figure S3). The heterogeneity was insignificant (*p* = 1.00, *I* ^2^ = 0%).

#### Immunoglobulins

**FIGURE 7:**
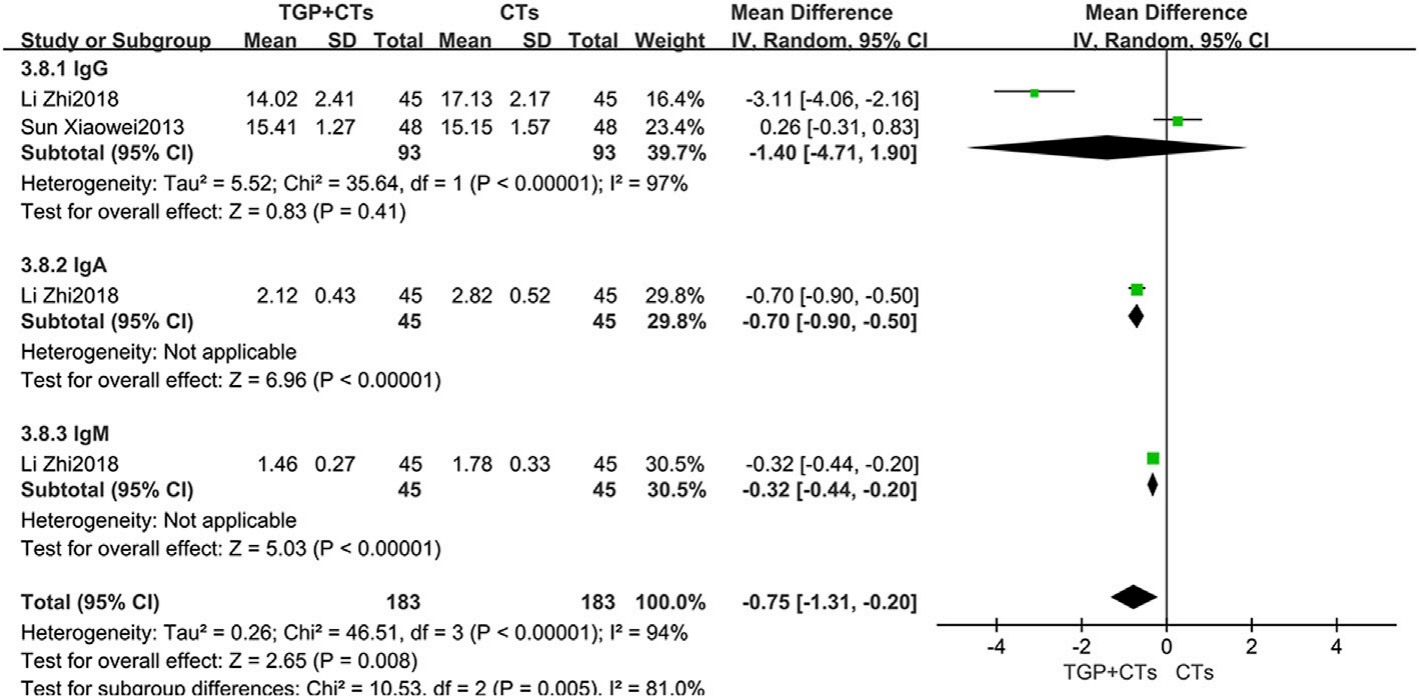
Forest plot of IgA, IgM and IgG.

Two trials reported immunoglobulins, and the results of single trial showed that TGP plus CTs was superior to CTs alone in reducing IgA and IgM (*MD*_IgA_ = −0.70, 95% *CI* = -0.90 to −0.50, *p* < 0.00001; *MD*_IgM_ = −0.32, 95% *CI* = −0.44 to −0.20, *p* < 0.00001; Figure 7). However, the pooled results of two trials showed that there were no statistical difference in reducing IgG between two groups (*MD*_IgG_ = −1.40, 95% *CI* = −4.71 to 1.90, *p* = 0.41; Figure 7).

#### Average Daily Glucocorticoid Dosage

**FIGURE 8:**
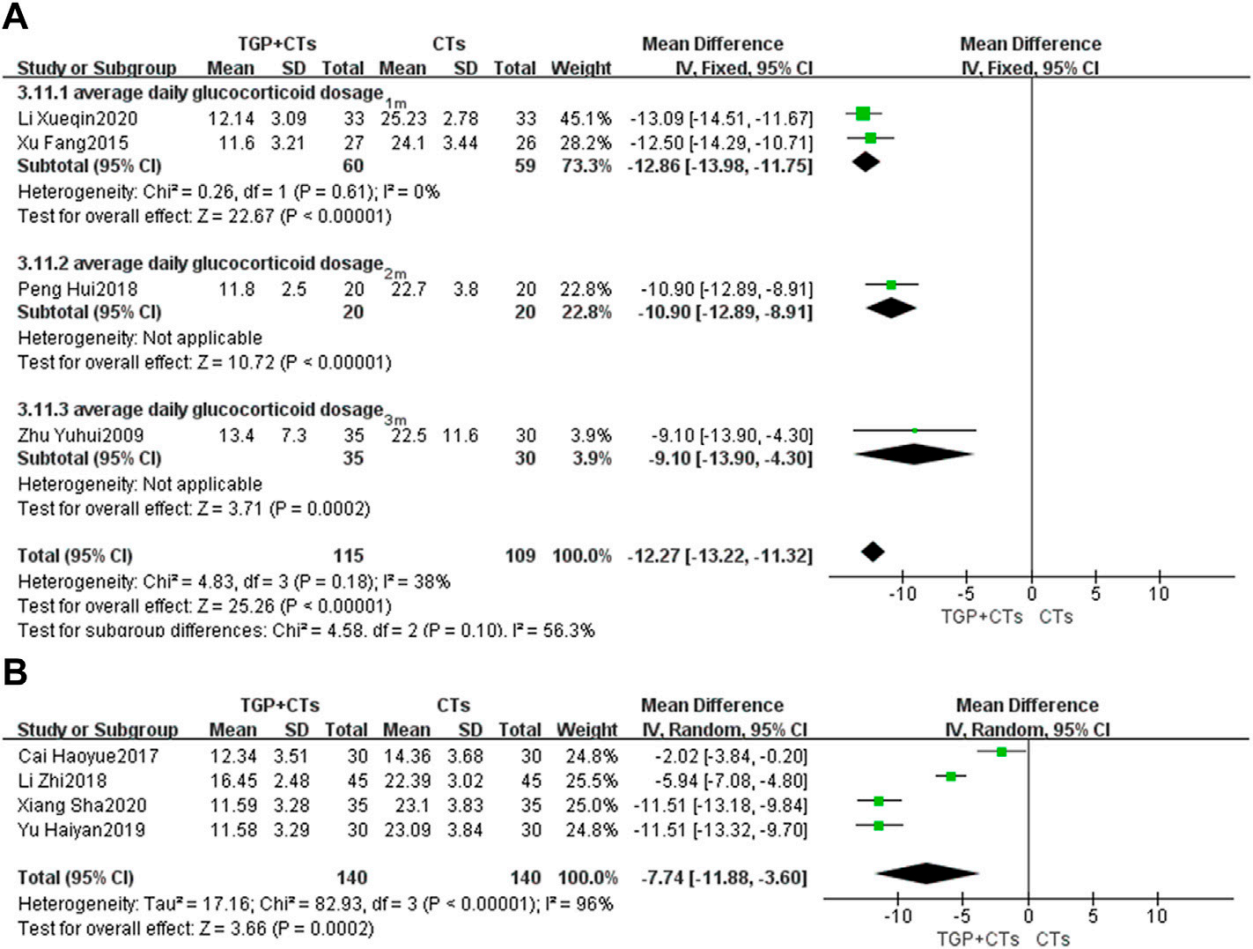
Forest plot of average daily glucocorticoid dosage at 1 month, 2 and 3 months **(A)** and 6 months **(B)**.

Eight trials reported average daily dosage of glucocorticoid. We performed a subgroup analysis based on treatment duration. According to different heterogeneity (*P*_1m_ = 0.61, *I* ^2^ = 0%; *P*_6m_ < 0.00001, *I* ^2^ = 96%), we used different effect models. The results showed that TGP plus CTs was superior to CTs alone in reducing average daily glucocorticoid dosage at one, two and 3 months (*MD* = −12.27, 95% *CI* = −13.22 to −11.32, *p* < 0.00001; Figure 8A). There was also a statistical difference between two groups in reducing average glucocorticoid dosage at 6 months (*MD* = −7.74, 95% *CI* = −11.88 to −3.60, *p* = 0.0002; Figure 8B).

However, the heterogeneity of 6 months subgroup was large, we conducted sensitivity analysis and found that the two trials (Cai et al., 2017; Li et al., 2018) and other trials had clinical heterogeneity in terms of administration frequency and dose. We removed the two trials, and pooled other trials (*MD* = -11.51, 95% *CI* = −12.74 to −10.28, *p* < 0.00001; Supplementary Figure S4). The heterogeneity was insignificant (*p* = 1.00, *I* ^2^ = 0%).

#### Cumulative Cyclophosamide Dosage

**FIGURE 9:**
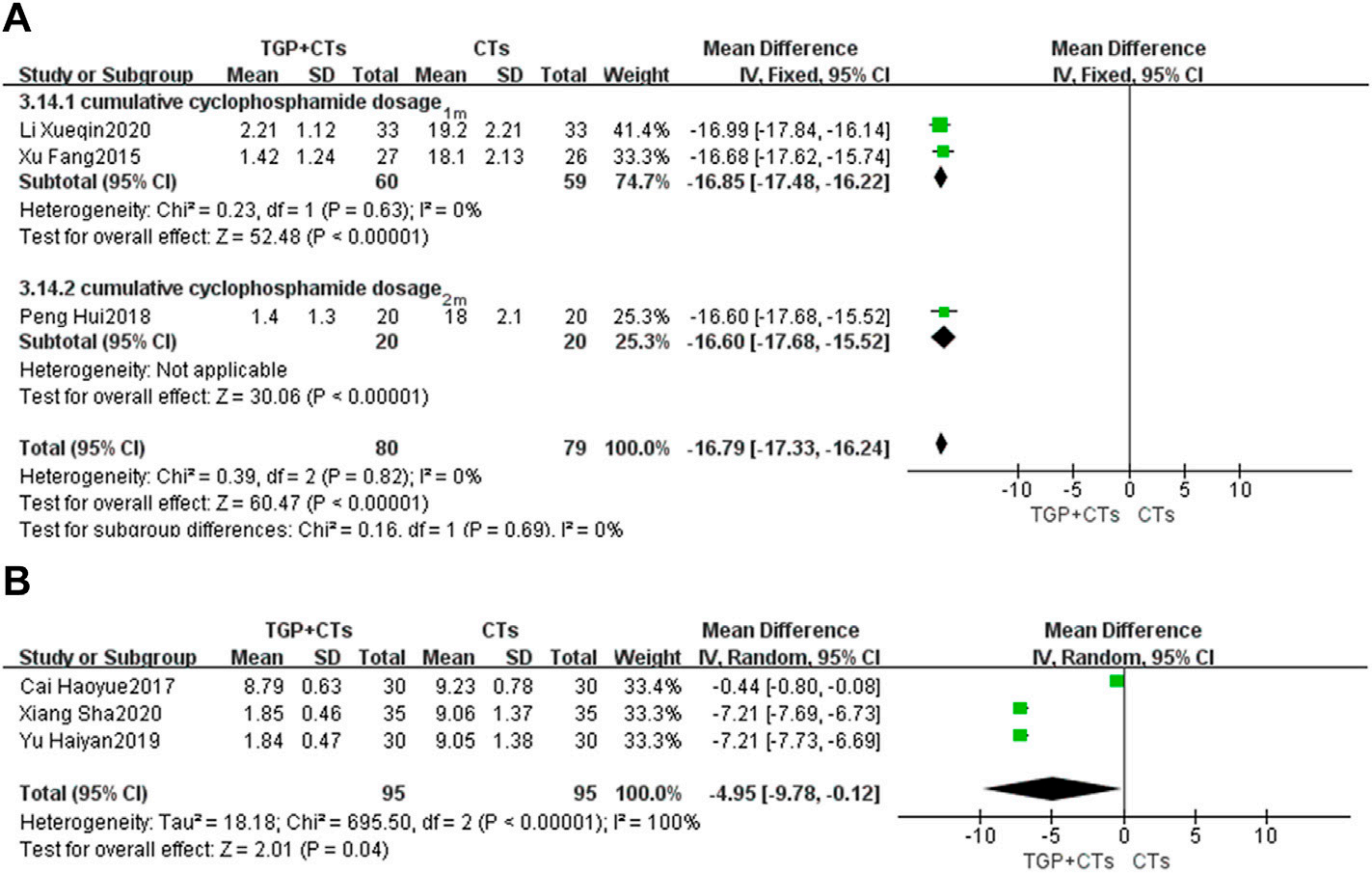
Forest plot of cumulative cyclophosamide dosage at 1 month, 2 months **(A)** and 6 months **(B)**.

Six trials reported cumulative cyclophosamide dosage. We performed the subgroup analysis based on treatment duration. According to different heterogeneity (*P*_1m_ = 0.63, *I* ^2^ = 0%; *P*_6m_ < 0.00001, *I* ^2^ = 100%), we used different effect models. The results showed that TGP plus CTs was superior to CTs alone in reducing cumulative cyclophosamide dosage at one and 2 months (*MD* = −16.79, 95% *CI* = −17.33 to −16.24, *p* < 0.00001; Figure 9A). There was also a statistical difference between two groups in reducing cumulative cyclophosamide dosage at 6 months (*MD* = −4.95, 95% *CI* = −9.78 to −0.12, *p* = 0.004; Figure 9B).

Because of the substantial heterogeneity of 6 months subgroup, we carried out sensitivity analysis but found no potential clinical heterogeneity or methological heterogeneity by reading original literatures.

#### Disease Recurrence Rate

**FIGURE 10:**
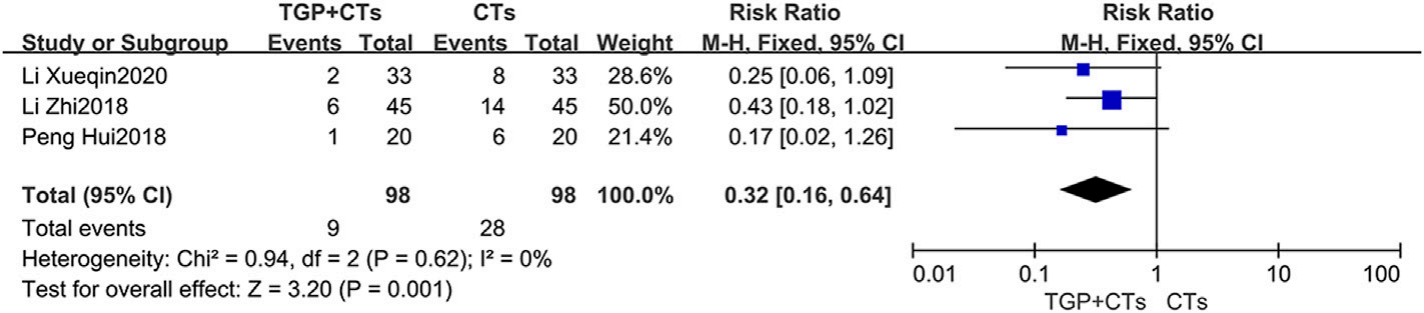
Forest plot of disease recurrence rate.

Three trials reported recurrence rate, with no significant heterogeneity among trials (*p* = 0.68, *I* ^2^ = 0%), and the fixed effect model was used. The results demonstrated that TGP plus CTs showed a weighty decrease on the recurrence rate of SLE compared with CTs alone (*RR* = 0.32, 95% *CI* = 0.16 to 0.64, *p* = 0.0009; Figure 10).

### Safety Outcomes

#### Incidence of Adverse Reactions

**FIGURE 11:**
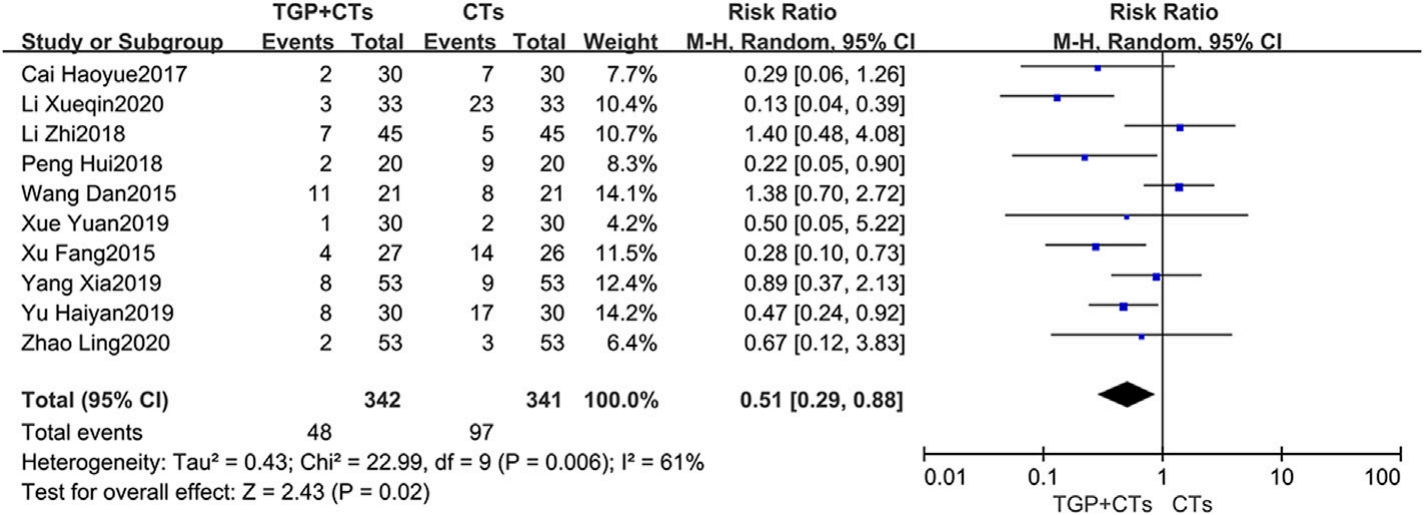
Forest plot of incidence of adverse reactions.

Adverse reactions were reported in ten trials, with large heterogeneity (*p* = 0.002, *I* ^2^ = 66%), and random effect model was used. Incidence of adverse reactions occurred in 48 out of 342 patients (14.0%) who received TGP plus CTs and 97 out of 341 patients (28.4%) who received CTs alone. The results showed that the incidence of adverse reactions in TGP group was significantly lower than control group, with a statistical difference (*RR* = 0.51, 95% *CI* = 0.29–0.88, *p* = 0.01; Figure 11).

The reported adverse reactions in two groups included infection (pulmonary infection, urinary tract infection, fungal infection), gastrointestinal reaction, osteoporosis, leucopenia, dizziness, insomnia, fever and acne.

#### Adverse Events

None of the trials reported adverse events.

#### Publication Bias

**FIGURE 12:**
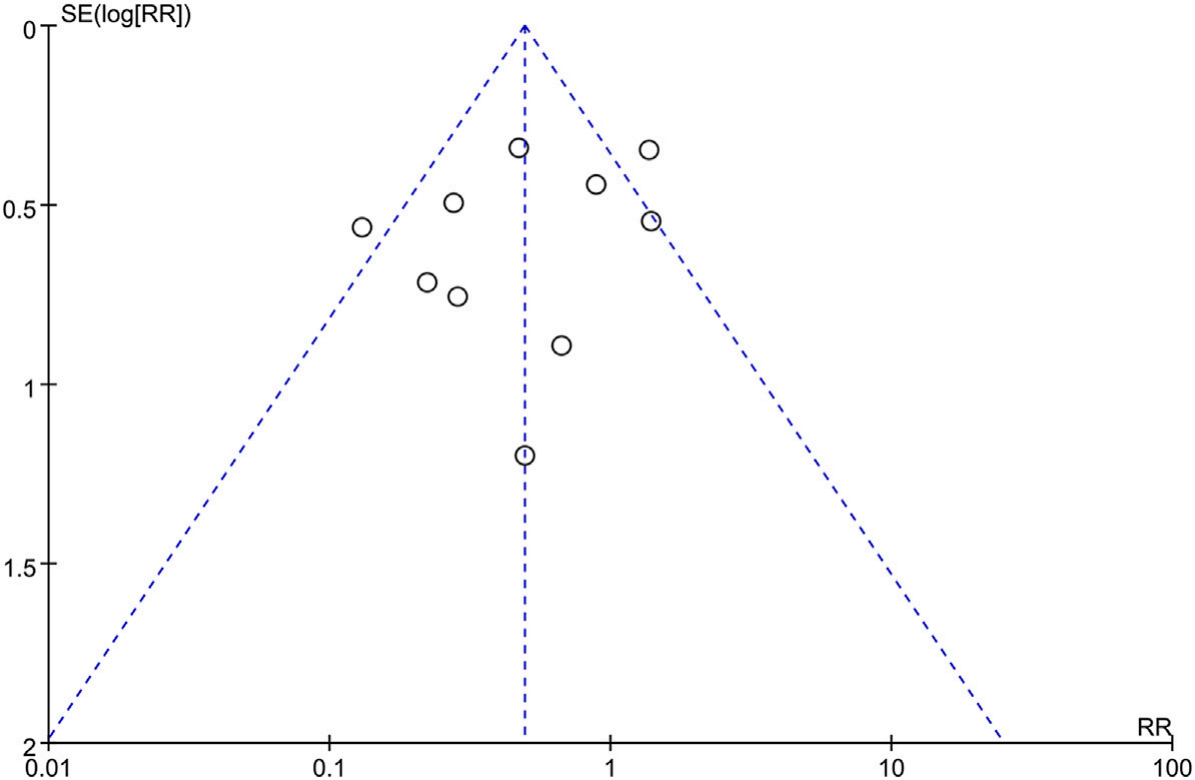
Funnel plot of incidence of adverse reactions.

**FIGURE 13:**
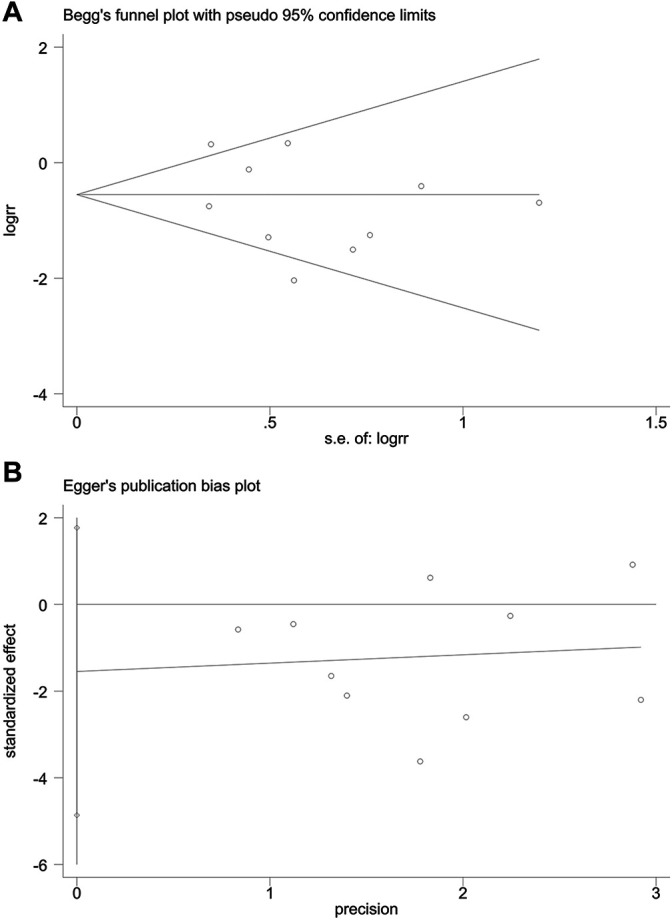
Publication bias. **(A)** The Begg’s test on incidence of adverse reactions. **(B)** The Egger’s test on incidence of adverse reactions.

The publication bias of incidence of adverse reactions was evaluated by the funnel plot (Figure 12). The Begg’s test (Figure 13A) and Egger’s test (Figure 13B) showed that the *p* value was all greater than 0.05 (Begg, *p* = 0.721; Egger, *p* > 0.313), which indicated that the publication bias of incidence of adverse reactions was insignificant.

#### Sensitivity Analysis

We carried out sensitivity analysis for all outcomes to investigate the robustness of results. After removing trials one by one, we found no change in the statistical difference of results, which demonstrated that our results were robust. The forest plots of sensitivity analysis regarding exploring the heterogeneity are shown in Supplementary Material.

#### GRADE Assessment

We choosed seven outcomes (SLEDAI-1m, SLEDAI-3m, SLEDAI-6m, incidence of adverse reactions, disease recurrence rate, ESR and 24-h urine protein), and assessed the certainty of evidence as “moderate,” or “low,” using the GRADE system. The main reasons of downgrading were high risk of bias and inconsistency. The GRADE evidence profiles are shown in Table 2.

**TABLE 2:** GRADE summary of outcomes for TGP + CTs versus CTs for patients with SLE.

Outcomes	No. of participants (studies)	Anticipated absolute effects (95% *CI*)		Relative effect (95% *CI*)	Certainty of the evidence (GRADE)
		Risk with CTs	Risk difference with TGP + CTs		
SLEDAI-1m	119 (2)	The mean SLEDAI-1m ranged from 5.73 to 5.9	The mean SLEDAI-1m in the TGP + CTs group was 3.54 lower (4.08 lower to 3 lower)	-	⊕⊕⊕○ Moderate^a^
SLEDAI-3m	415 (5)	The mean SLEDAI-3m ranged from 4.71 to 16	The mean SLEDAI-3m in the TGP + CTs group was 1.62 lower (2.6 lower to 0.64 lower)	-	⊕⊕○○ LOW^a^ ^,^ ^b^
SLEDAI-6m	344 (5)	The mean SLEDAI-6m ranged from 2.88 to 6.17	The mean SLEDAI-6m in the TGP + CTs group was 1.97 lower (3.18 lower to 0.76 lower)	-	⊕⊕○○ LOW^a^ ^,^ ^b^
Incidence of Adverse Reactions	683 (10)	284 per 1,000	145 fewer per 1,000 (182 fewer to 94 more)	*RR* 0.49 (0.36–0.67)	⊕⊕○○ LOW^a^ ^,^ ^b^
Disease Recurrence Rate	196 (3)	286 per 1,000	194 fewer per 1,000 (240 fewer to 103 more)	*RR* 0.32 (0.16–0.64)	⊕⊕⊕○ Moderate^a^
ESR	245 (4)	The mean ESR ranged from 25.67 to 45.98	The mean ESR in the TGP + CTs group was 7.68 lower (9.12 lower to 6.25 lower)	-	⊕⊕⊕○ Moderate^a^
24-Hour Urine Protein	315 (5)	The mean 24-Hour Urine Protein ranged from 0.66 to 1.88	The mean 24-Hour Urine Protein in the TGP + CTs group was 0.37 lower (0.64 lower to 0.09 lower)	*RR* 1.20 (1.16–1.24)	⊕⊕○○ LOW^a^ ^,^ ^b^

TGP, total glucosides of paeony; CTs, conventional treatment; CI, confidence interval; RR, relative risks.

a Poor methodology including method of randomization, allocation concealment and blinding.

b *I* ^2^ ≥ 50% for heterogeneity.

## Discussion

### Summary of Findings

In this systematic review, fourteen RCTs with 978 participants were included, and the results of meta-analysis showed that TGP effectively and safely reduced the disease activity, with low to moderate certainty of evidence. For the primary outcome, TGP improved SLEDAI score during 1–6 months, but the effect size in the early stage is more significant with higher quality evidence. A retrospective study found that the SLEDAI of SLE patients continuously taking TGP for 5 years was significantly lower than that of the patients intermittently taking TGP and those not taking it (Zhang et al., 2011). Therefore, the efficacy of TGP is definite but slow. For laboratory outcomes, the results showed positive effects of TGP on improving ESR, CRP, C3, C4, IgA, IgM and 24-h urine protein. Although ESR is a non-specific parameter, it appears to be a reliable marker for disease activity assessment in non-infected SLE patients (Dima et al., 2016). The overall increase of CRP baseline in SLE is not followed by an increase up to the level during flares, but CRP is related to infections and risk of cardiovascular events in SLE (Gheita et al., 2012; Fakhreldin et al., 2015). As monitoring disease activity, C3 and C4 can decrease prior to a clinically evident flare (Petri et al., 2013). The urine proteins directly reflect renal pathology and 24-h urine protein was positively correlated with the activity of lupus (Li et al., 2018). For safety outcomes, the results showed that TGP as an adjuvant therapy greatly inhibited the adverse reactions of CTs.

### Strengths and Limitations

This is the first systematic review reporting the certainty of evidence of TGP in reducing SLE activity, rigorously following the GRADE approach and the PRISMA statement. Our review also has limitations. Firstly, safety assessment was inadequate. The potential causal relationship between adverse reactions and TGP was not evaluated. Few studies reported the ocurrance time and severity of adverse reactions such as fever, gastrointestinal reactions and infection. Adverse reactions are likely to be specific, but the individual characteristics of the subjects with adverse reactions were not fully reported. Therefore, the uncertainty of safety profile of TGP was noticeable. Another safety issue was that no trials reported the adverse events. The reason may be the confusion about concepts of adverse reactions and adverse events. The former has definite relationship with the use of normal dose drugs, while the latter also includes other unfavorable events with no definite relationship. The lack of judgment on causality for adverse events induced potential bias of selective reporting. Secondly, there was an evaluation gap from disease activity to quality of life (QoL), the final endpoint. Although some studies have shown that lupus low disease activity state was associated with improved QoL (Poomsalood et al., 2019; Louthrenoo et al., 2020), nevertheless in many patient’s perspective QoL and fatigue were insufficient controlled in low disease activity (Kernder et al., 2020). Moreover, the absence of subjective manifestations is a shortcoming of SLEDAI. Thirdly, the poor methodology of included trials affected the reliability of results. Included trials generally lacked a description of allocation concealment, which induced a high risk of selection bias. Only by combining randomization with blinding can we really control the risk of bias. However, none of included trials reported blinding, which induced performance bias and detection bias. The above biases exaggerated results and reduced the reliability of results (Wang et al., 2014; Mitchell et al., 2020). In addition, we were unable to investigate the time-response and dose-response relationships, owing to the short course of treatment and the low number of trials.

### Implications for Future Research

In the evaluation of TGP, safety should always take precedence over efficacy. Especially for SLE therapies, the safety comparison among different therapeutic drugs is the first and common concern of patients and doctors. According to the current limited evidence, as an adjuvant therapy, TGP were likely to reduce toxicity by gradually decreasing the dosage of GC and CTX. In addition to adverse reactions, researchers should also record and report adverse events in detail. The safety evaluation of TGP deserves attention and further improvement. Patient-oriented trials are essential to investige whether TGP can not only reduce the lupus activity, but also improve the QoL of lupus patients. Up to date, the QoL is not the primary outcome, but a necessary part of subjective perception of patients nevertheless (Olesińska and Saletra, 2018). For the measurement tool of lupus activity, SLEDAI-2K, allowing for persistent activity, is more suitable than the original SLEDAI for assessment of global disease activity in SLE. Rigorous designed trials are urgently needed to upgrade the quality of evidence in TGP reducing lupus activity. TGP, different from traditional adjuvant therapies, can contribute to the low dosage of CTs. Accordingly, TGP has the potential to become a promising alternative therapy for SLE. Long term efficacy should be explored in the future.

## Conclusion

Moderate or low certainty evidence demonstrated that TGP had excellent efficacy on reducing lupus activity. However, the evidence on safety of TGP for SLE was insufficient. More strong evidence for clinical practice still requires large-scale and high-quality RCTs.

## Data Availability

The original contributions presented in the study are included in the article/Supplementary Material, further inquiries can be directed to the corresponding author.
