# Supplementary material for: Total Glucosides of Paeonia lactiflora for Safely Reducing Disease Activity in Systemic Lupus Erythematosus: A Systematic Review and Meta-Analysis
Source: Front Pharmacol. 2022 Jan 31;13:834947. doi: 10.3389/fphar.2022.834947 (PMC8841895; doi:10.3389/fphar.2022.834947)
Supplement: Supplementary file 2 [file DataSheet1.docx]

Supplementary Material

# Supplementary Figures and Tables

## Supplementary Figures

##
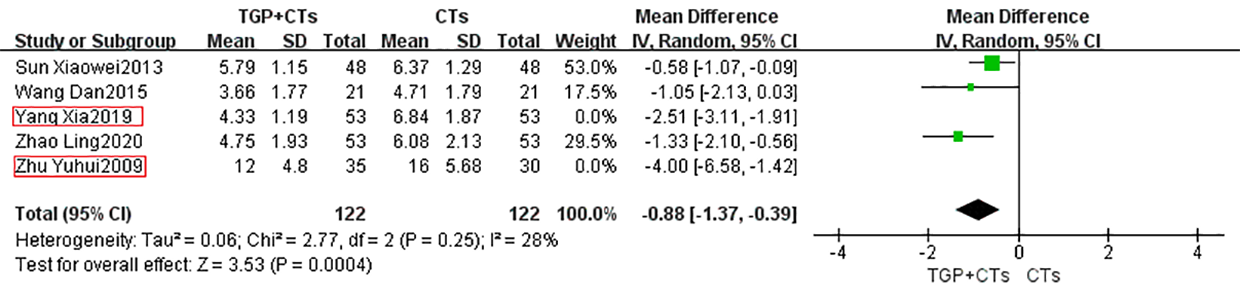
Supplementary Figure 1. Sensitivity analysis of SLEDAI-3m.

##
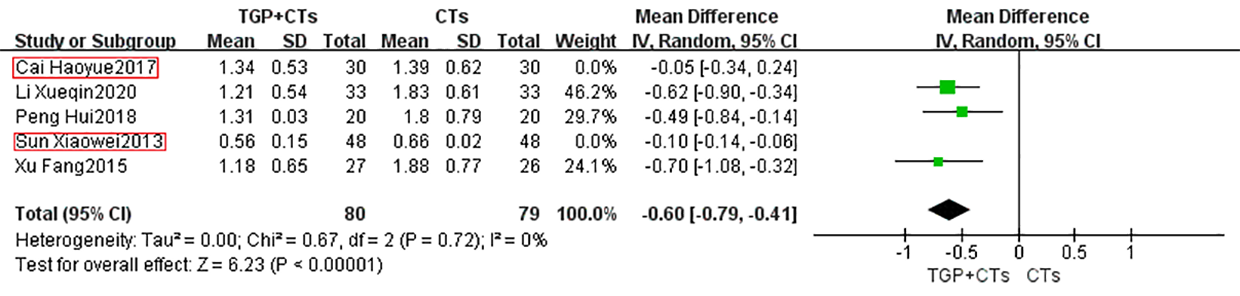


## Supplementary Figure 2. Sensitivity analysis of 24-hour urine protein.


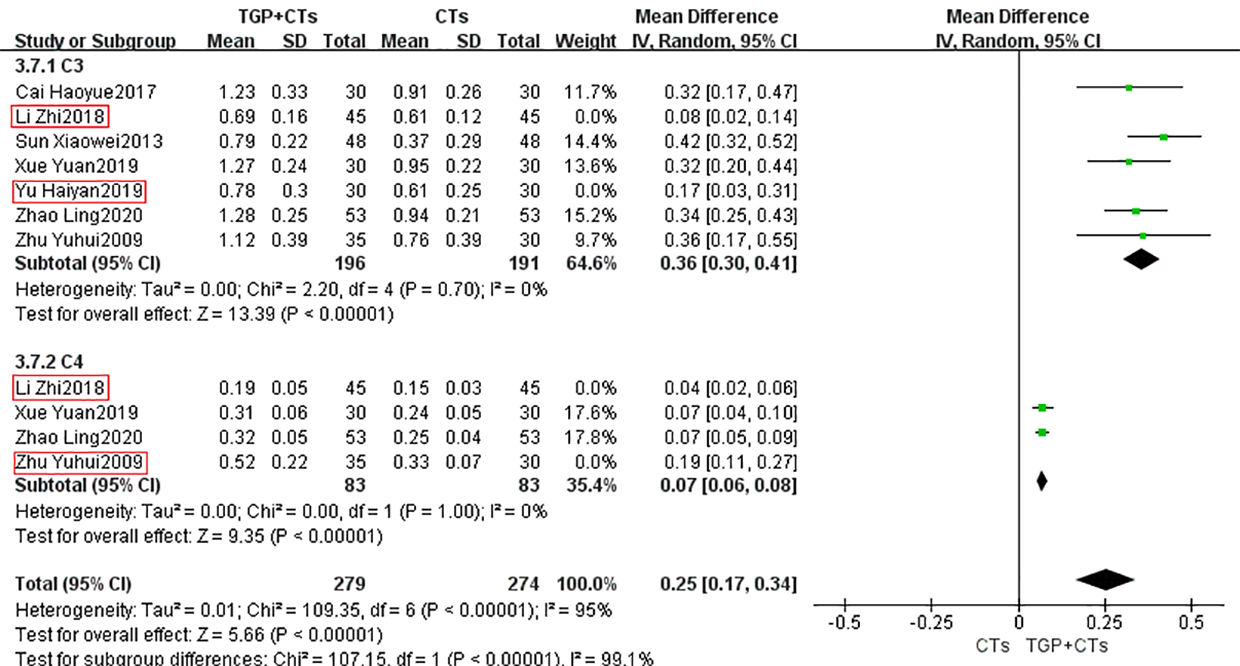


**Supplementary Figure 3.** Sensitivity analysis of C3 and C4.


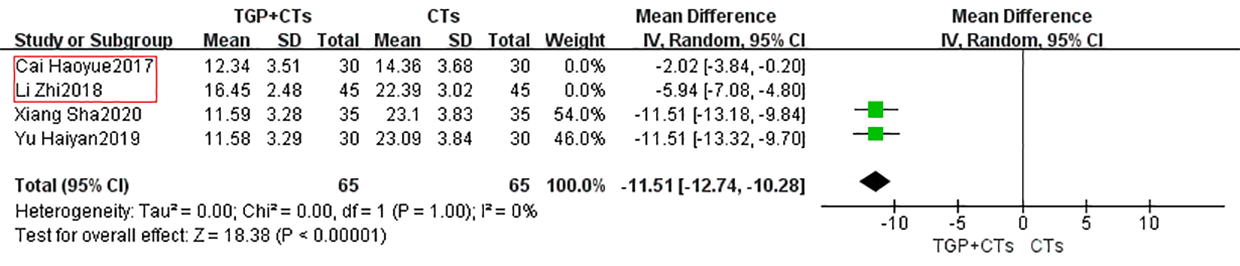


**Supplementary Figure 4.** Sensitivity analysis of average daily glucocorticoid dosage at 6 months.

## Supplementary Tables

**Supplementary Table 1.** The search strategies for all databases.

| **The search strategy for PubMed** | |
| --- | --- |
| **Number** | **Search terms** |
| #1 | Lupus Erythematosus, Systemic[MeSH Terms] |
| #2 | ((((Lupus Erythematosus, Systemic[Title/Abstract]) OR (Systemic Lupus Erythematosus[Title/Abstract])) OR (Lupus Erythematosus Disseminatus[Title/Abstract])) OR (lupus[Title/Abstract])) OR (sle[Title/Abstract]) |
| #3 | #1 OR #2 |
| #4 | ((Total Glucosides of Paeony[Title/Abstract]) OR (TGP[Title/Abstract])) OR (Paeoniflorin[Title/Abstract]) |
| #5 | #3 AND #4 |
| **The search strategy for Embase** | |
| **Number** | **Search terms** |
| #1 | exp systemic lupus erythematosus/ |
| #2 | exp lupus erythematosus/ |
| #3 | (lupus or sle).tw. |
| #4 | 1 or 2 or 3 |
| #5 | (total glucosides paeony or TGP or Paeoniflorin).tw. |
| #6 | 4 and 5 |
| **The search strategy for Cochrane Library** | |
| **Number** | **Search terms** |
| #1 | MeSH descriptor: [Lupus Erythematosus, Systemic] explode all trees |
| #2 | (lupus):ti,ab,kw OR (sle):ti,ab,kw |
| #3 | (total Glucosides of paeony):ti,ab,kw OR (TGP):ti,ab,kw OR (Paeoniflorin):ti,ab,kw |
| #4 | #1 OR #2 |
| #5 | #3 AND #4 |
| **The search strategy for CNKI** | |
| (SU = '系统性红斑狼疮' OR SU = '系统红斑狼疮' OR SU = '红蝴蝶疮') AND (SU= '白芍总苷' OR SU = '白芍总甙') | |
| **The search strategy for WanFang** | |
| 主题: ("系统性红斑狼疮"+"系统红斑狼疮"+"红蝴蝶疮") and 主题: ("白芍总苷"+"白芍总甙") | |
| **The search strategy for VIP** | |
| ((M=系统性红斑狼疮 OR 系统红斑狼疮 OR 红蝴蝶疮) OR (R=系统性红斑狼疮 OR 系统红斑狼疮 OR 红蝴蝶疮)) AND ((M=白芍总苷 OR 白芍总甙) OR (R=白芍总苷 OR 白芍总甙)) | |
| **The search strategy for CBM** | |
| #1 | "系统性红斑狼疮"[常用字段:智能] OR "系统红斑狼疮"[常用字段:智能] OR "红蝴蝶疮"[常用字段:智能] |
| #2 | "白芍总苷"[常用字段:智能] OR "白芍总甙"[常用字段:智能] |
| #3 | #1 AND #2 |

**Supplementary Table 2.** The list of excluded reports.

| **Report excluded** | **Reason** |
| --- | --- |
| Feng Pingzhen 2017 | Wrong data |
| Yang Maoji 2016 | Unclear course of treatment |
| Xu Yan 2020 | Unclear course of treatment |
| Lin Guoyou 2016 | Unclear course of treatment |
| Liu Wei 2016 | Unclear course of treatment |
| Li Yanmei 2013 | Unclear course of treatment |
| Chen Jing 2013 | Unclear course of treatment |
| Wang Zhenjie 2013 | Unclear course of treatment |
| Shuai Zongwen2003 | Large clinical heterogeneity |

**References**

Chen, J. (2013). Therapeutic effect and side effects of total glucosides of paeony on systemic lupus erythematosus. China Healthcare Innovation. 8(06), 28+7. doi: CNKI:SUN:YLQY.0.2013-06-019

Feng, P. Z., Ji, H. l., and Li, Y. L. (2017). Clinical effect of cyclophosphamide combined with total glucosides of paeony in the treatment of systemic lupus erythematosus. Laboratory Medicine and Clinic. 14(z2). doi: 10.3969/j.issn.1672-9455.2017.26.049

Li, Y. M. (2013). Clinical effect of total glucosides of paeony on systemic lupus erythematosus. Journal of clinical rational drug use. 6(22), 68-69. doi: 10.15887/j.cnki.13-1389/r.2013.22.014

Liu, W. (2016). Clinical effect of total glucosides of paeony on systemic lupus erythematosus. Chinese Journal of Modern Drug Application. 10(04), 144-145. doi: 10.14164/j.cnki.cn11-5581/r.2016.04.109

Lin, G. Y., and Liu, C. X. (2016). To observe the therapeutic effect and side effects of total glucosides of paeony on systemic lupus erythematosus. Journal of North Pharmacy. 13(02), 155. doi: CNKI:SUN:BFYX.0.2016-02-124

Shuai, Z. W., Xu, J. H., Liu, S., Wei, W., Xu, S. Q., and Xu, S. Y. (2003). Clinical observation of total glucosides of paeony in the adjuvant treatment of systemic lupus erythematosus. Chinese Journal of Integrated Traditional and Western Medicine (03), 188-191. doi: CNKI:SUN:ZZXJ.0.2003-03-011

Wang, Z. J., Wang, J., and Liu, L. P. (2013). Clinical effect of total glucosides of paeony on systemic lupus erythematosus. Zhejiang clinical medicine (3). doi: 10.3969/j.issn.1008-7664.2013.03.036

Xu, Y. (2020). Clinical efficacy and safety of cyclophosphamide combined with total glucosides of paeony in the treatment of systemic lupus erythematosus. Guide of China Medicine. 18(10), 83-84. doi: 10.15912/j.cnki.gocm.2020.10.036

Yang, M. J. (2016). To study the efficacy of cyclophosphamide combined with total glucosides of paeony in the treatment of systemic lupus erythematosus. Psychological Doctor. 22(24)

**Supplementary Table 3.** Botanical.

| **Study** | **Species, source, concentration** | **Quality control reported**  **(Y/N)** | **Chemical characterisation of the preparation** |
| --- | --- | --- | --- |
| Zhu Yuhui 2009 | - Dried roots of *Paeonia lactiflora* Pall., [NA], concentration uncertainty | N | Total Glucosides of Paeony  1.Paeoniflorin:  immunoregulatory, anti-inflammatory, antioxidant, analgesia;  2.Albiflorin:  anti-inflammatory, analgesia;  3. Benzoyl paeoniflorin;  4. hydroxy-paeoniflorin;  5. paeonin; |
| Sun Xiaowei 2013 | - Dried roots of *Paeonia lactiflora* Pall., [Sanjiu Pharmaceutical Co., Ltd], concentration uncertainty | Y –National medicine permission number: H19980147 |  |
| Wang Dan  2015 | - Dried roots of *Paeonia lactiflora* Pall., [Sanjiu Pharmaceutical Co., Ltd], concentration uncertainty | N |  |
| Xu Fang  2015 | - Dried roots of *Paeonia lactiflora* Pall., [Ningbo Lihua Pharmaceutical Co., Ltd], concentration uncertainty | Y –National medicine permission number: H20055058 |  |
| Cai Haoyue  2017 | - Dried roots of *Paeonia lactiflora* Pall., [Ningbo Lihua Pharmaceutical Co., Ltd], concentration uncertainty | Y –National medicine permission number: H20055058 |  |
| Li Zhi 2018 | - Dried roots of *Paeonia lactiflora* Pall., [Ningbo Lihua Pharmaceutical Co., Ltd], concentration uncertainty | Y –National medicine permission number: H20055058 |  |
| Peng Hui 2018 | - Dried roots of *Paeonia lactiflora* Pall., [NA], concentration uncertainty | N |  |
| Yang Xia  2019 | - Dried roots of *Paeonia lactiflora* Pall., [Ningbo Lihua Pharmaceutical Co., Ltd], concentration uncertainty | Y –National medicine permission number: H20055058 |  |
| Xue Yuan  2019 | - Dried roots of *Paeonia lactiflora* Pall., [Ningbo Lihua Pharmaceutical Co., Ltd], concentration uncertainty | Y –National medicine permission number: H20055058 |  |
| Yu Haiyan  2019 | - Dried roots of *Paeonia lactiflora* Pall., [Ningbo Lihua Pharmaceutical Co., Ltd], concentration uncertainty | Y –National medicine permission number: H20055058 |  |
| Zhang Yi  2020 | - Dried roots of *Paeonia lactiflora* Pall., [NA], concentration uncertainty | N |  |
| Zhao Ling  2020 | - Dried roots of *Paeonia lactiflora* Pall., [Ningbo Lihua Pharmaceutical Co., Ltd], concentration uncertainty | Y –National medicine permission number: H20055058 |  |
| Xiang Sha 2020 | - Dried roots of *Paeonia lactiflora* Pall., [NA], concentration uncertainty | N |  |
| Li Xueqin  2020 | - Dried roots of *Paeonia lactiflora* Pall., [Ningbo Lihua Pharmaceutical Co., Ltd], concentration uncertainty | Y –National medicine permission number: H20055058 |  |
